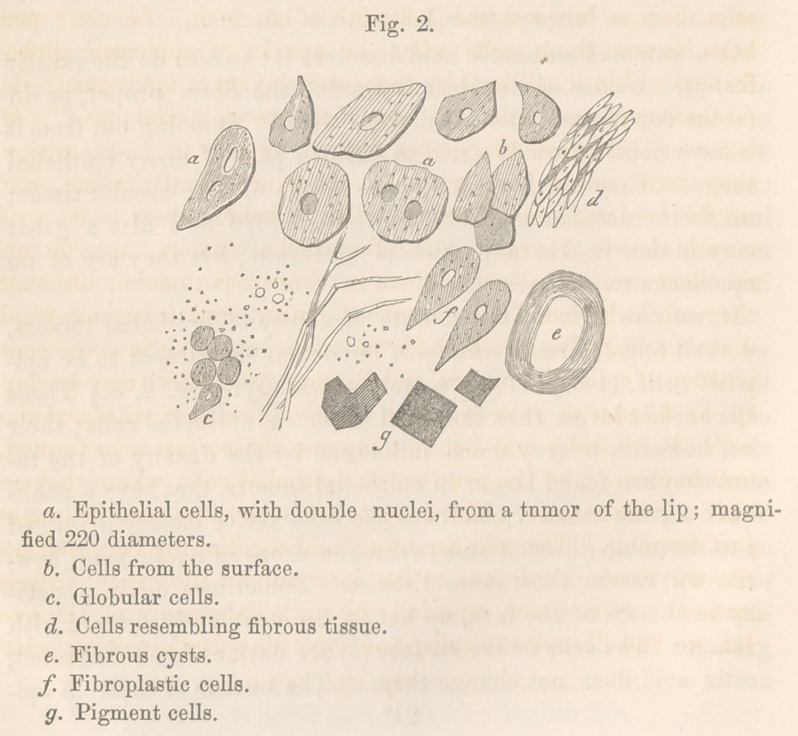# Remarks on Epithelial Tumors and Cancer of the Skin

**Published:** 1852-04

**Authors:** J. Da Costa

**Affiliations:** Philadelphia


*Remarks on Epithelial Tumors and Cancer of the Skin.* By J. Da Costa, M. D., of Philadelphia.

Anything thing that might elucidate, or be in any way connected with the histology of morbid growths, especially with that of cancer, has been of late of so much interest to us, that it is astonishing, so few have noticed the evident difference, existing between epithelial tumors, and cancerous affections of the skin. All writers on morbid anatomy, who have at all alluded to them, have classed them under one head—cancer—without paying the least regard to their different microscopical characters, or to the different results, following the same treatment employed. The fact, however, of most of these tumors not returning, led some surgeons to propose the term local or epithelial cancer; and induced one or two modern pathologists, a few years later, to investigate this disease. The first of these was Ecker, the next Mayor,* who, although correct in some respects, fell into the other extreme, endeavoring to prove, that although all these tumors were cancerous, on account of their tendency to return, yet they assumed entirely the epithelial element, and that this formed the cancer in every instance. In this opinion he was supported for some time by the most eminent living pathologist, Rokitansky, who, p. 385, Vol. 1st, of his Handbuch der allgemeinen pathologischen Anatomie, says: "Epidermic cancers are like medullary cancer, only having epithelial cells as their constituents." From careful microscopical observation, however, we find that most of these tumors are really epithelial, only an hypertrophy of epidermis or epithelium, whilst others, entirely overlooked by Mayor and Rokitansky, have another element, and are cancerous. Lebert in the best, and in many respects, the only complete paper† recently published, has made this the basis of his division, and following the nomenclature of Bennett, divides tumors of the skin into cancroid and cancerous. This was the great standard to establish, and one to which every observer must at last come. I shall, therefore, follow Lebert's division, and speak of tumors of the

* Thèse sur les tumeurs épidermiques et leurs relations avec l'affection cancéreuse. Paris, 1846.

† Du Cancer et du cancroïde de la Peau. Paris, 1851.

skin, as epithelial or epidermic, and as cancerous tumors; not adopting the word cancroid for epithelial, but calling those epithelial or epidermic, that have no cancerous element. As epidermis is, however, epithelium, we might designate the tumors on the skin, that more strictly ought to be called epidermic, epithelial; and those cancerous, which are really cancer; avoiding the use of the word epithelial cancer, which has led to so much confusion, and which ought never to be used. I have examined about 40 cases, occurring on the lip, face, body, gums, and tongue; as they all presented nearly the same elements, and their treatment could be reduced to the same principles, I shall give but a general description, although, did the limits of this paper allow, I should have preferred noticing individually each part of the body, where they occur.

*Epithelial Tumors proper*

Constitute the majority of all cutaneous tumors, and are mainly but an hypertrophy of healthy tissue. They occur on the face, there constituting the so called noli me tangere, on the lip, trunk, back of hand, foot, gums and tongue. Most frequently do we meet with them in the lower lip. Bock mentions a case of an epithelial tumor being found in the conjunctiva; I have examined one that sprung from the knee joint. Out of 25 cases I have noted down, there were

| | |
|---|---|
| Epithelial tumor of lip . . . . | 11 |
| " " face . . . . . | 7 |
| " " trunk . . . . | 1 |
| " " extremities . . . | 2 |
| " " penis . . . | 1 |
| " " tongue . . . | 2 |
| " " gums . . . | 1 |
| Total, | 25 |

According to Lebert, these tumors, when they first appear, assume three forms; hypertrophy of the epidermis alone, hypertrophy of the papillæ, and hypertrophy with infiltration into the surrounding tissue. Belonging in some respects to the first variety, are a great many horny excrescences, some forms of corns, and other morbid growths, that result from continued pressure. The papillæ, when they become hypertrophied, form one of the most singular constituents of epidermic tumors; they are found in many simple warts. The third form, which, extending to the muscles, can even infect the neighboring lymphatics, is usually the worst and last stage of development of epithelial formations. All these varieties are mixed in tumors we commonly meet with, for even those, which begin merely as hypertrophy of the epidermis, often terminate by infiltration into the surrounding tissue. These tumors, after they have been completely developed, exhibit a strange tendency to ulcerate; a tendency which is most marked in those on the lip and face, where they ulcerate, forming sometimes a smooth superficial, sometimes a reddish ulcer. The ulceration is in no case deep, but rather extends in length, begins from the surface, and not as in real cancer, from the deepest part of the tumor.

The general appearance of these tumors varies in different parts of the body; they all are elevated above the surrounding tissue, reddish on their surface, and very apt to inflame the neighboring parts. On the lip, they present either the appearance of a ripe mulberry, or a peculiar tuberculated form, and have a dark red crust on their exterior. On the face, they usually are about the size of a dime, and very red. In other parts of the body, they resemble in appearance very much the tumors of the lip; nearly all have on their surface, the same dark red crust.

Before describing their minute arrangement, we will examine the anatomy of the skin and mucous membrane, as far as they are important to our subject, and notice especially, the microscopical appearance of epithelial and epidermic cells. Epithelial cells are usually of three varieties; tesselated, cylindrical, and vibratile. Besides these, some of the French writers describe a form, between the tesselated and cylindrical, transitory epithelium, the "epithelium de transition." All these cells have several characteristics in common; it is only the shape of the cell-wall, that determines the variety to which they belong. They are all held together by an intercellular substance, which acetic acid dissolves. All of them, further, have nuclei, and contain granular matter, light in some cells, but very dark in others, and never larger than the 1-15000th part of an English inch. The nuclei vary in size in different parts of the body; to determine their relative size, in comparison, with those of the cells found in epithelial tumors, is a matter of interest, as will be shown hereafter. As the following measurements of Henle,* are acknowledged to be the only accurate ones, we have on this subject, I cannot do better than insert a condensed translation of his table.

| | | | | | |
|---|---|---|---|---|---|
| Nuclei of cells from the sole of the foot, | | | 0.0026 | of a Paris line† in diam. | |
| " | " | glans penis, | 0.0022 | " | " |
| " | " | tongue, 0.0020 | 0.0042 | " | " |
| " | " | deeper layer, 0.0011 | 0.0016 | " | " |
| " | " | mouth, 0.0030 | 0.0050 | " | " |
| " | " | peritoneum, | 0.0040 | " | " |
| " | " | mammary gland, | 0.0022 | " | " |
| " | " | vagina, | 0.0040 | " | " |
| " | " | nasal canal, | 0.0027 | " | " |
| " | " | trachea, | 0.0016 | " | " |
| " | " | choroid plexus, | 0.0025 | " | " |

The cells of tesselated epithelium are large, and have a pale nucleus, situated either in the middle, or at the inferior extremity of the cell. The large cells are pale, and not so granular as the small ones; they have elliptical nuclei, and sometimes nucleoli. (See fig. 1, *a.*) A variety of this tesselated epithelium, is that which lines arteries, lymphatics, veins and ducts; it is smaller and more oval. Some glands are lined by an epithelium, which is round and globular, and furnished with large nuclei. (*b.*)

Fig. 1.

*a.* Cells of tesselated epithelium, magnified 220 diameters.

*b.* Round epithelial cells lining glands.

*c.* Cylindrical epithelium from the trachea of a child; magnified 400 diameters.

*d.* Old epithelial cells.

*e.* Free nuclei.

* Anatomie Generale. Paris, 1843.

†One Paris line is the 4-45ths of an English line.

These cells are continually undergoing a change; the old ones die and are thrown to the surface. If we examine them in this stage, we find them dark, and with their edges curled up; some become perfectly fibrated, lose all characteristics of epithelial cells, and look like amorphous masses. (Fig. 1, *d.*) In these old cells, the nuclei are indistinct; while in the young ones, we have distinct granular nuclei. Cylindrical and vibratile epithelium (*c*) are similar; they have usually large nuclei. The transitory epithelium, is a variety between cylindrical and tesselated, sometimes seen in tumors of the lip. The cells have distinct nuclei, and frequently nucleoli. As regards the epidermis proper, it is tesselated epithelium. The cells, however, are in closer union, which is probably one of the reasons why tumors of the skin are denser in their structure, than those of the lip or gums.

It consists, according to Jahn, of

| | | |
|---|---|---|
| Horny matter, | 93 or 95 | parts. |
| Gelatinous matter, | 5 | " |
| Fatty granules, | 6.5 | " |
| Salts and acids, | 1.5 | " |

Concentrated sulphuric acid dissolves it; so also do the caustic alkalies. Under the epidermis we find the derm proper, or in mucous membranes, the sub-mucous tissue. Standing out from it we have papillæ, which form so large a part of many epithelial tumors. They are formed like the derma of fibro-areolar tissue; into their base run vessels and nerves. We find also a great many glands in skin and mucous membrane, but they are of no importance to us here.

If we now look at the minute structure of epithelial tumors, we shall find their first and most important constituent to be epithelial cells, chiefly of the tesselated variety. (Fig. 2, *a.*) These cells are not larger than the usual pavement epithelial cells; their size, however, is very much influenced by the density of the tumor; they are found larger in epithelial tumors, that have a cauliflower arrangement. Sometimes the cells are so closely arranged as to resemble fibrous tissue (*d*); a few drops of acetic acid, however, will render them more distinct. Sometimes again, especially in tumors of the face, we have them completely covered with granules. The cells on the surface (*b*) are dark and non-nucleated; acetic acid does not change them. The nuclei of cells in epithelial tumors are usually large and more distinct than those of common epithelial cells; many cells have double nuclei, (*a*) some nucleoli, but this is only dependent on most of these cells being more developed, and it will only be found to be so in tumors that have attained a large size. I measured accurately the nuclei of some cells from the internal surface of the lip, and some from the interior of an epithelial tumor of the lip; they were :

| HEALTHY LIP. | | TUMOR. | |
|---|---|---|---|
| Cell 1-800th of an inch, nucleus | 1-4000th | Cell 1-820th of an inch, nucleus | 1-3250th |
| Cell 1-1000th " " " | 1-4200th | Cell 1-1000th " " " | 1-3800th |
| Cell 1-2000th " " " | 1-5000th | Cell 1-1840th " " " | 1-4000th |
| Ceil 1-1820th " " " | 1-4800th | Cell 1-2500th " " " | 1-4400th |

But in a great many small tumors, especially those of the face, there was no difference at all.

| EPIDERMIS FROM FACE. | | TUMOR. | |
|---|---|---|---|
| Cell 1-200th of an inch, nucleus | 1-4900th | Cell 1-1890th of an inch, nucleus | 1-4950th |
| Cell 1-3000th " " " | 1-5820th | Cell 1-2980th " " " | 1-5600th |
| Cell 1-1820th " " " | 1-4400th | Cell 1-1800th " " " | 1-4500th |

Mixed up in all these tumors with the cells we find a great many free nuclei, crystals of cholesterin, pigment cells, (*g*) and

Fig. 2.

*a.* Epithelial cells, with double nuclei, from a tnmor of the lip; magnified 220 diameters.

*b.* Cells from the surface.

*c.* Globular cells.

*d.* Cells resembling fibrous tissue.

*e.* Fibrous cysts.

*f.* Fibroplastic cells.

*g.* Pigment cells.

blood globules. The second element we meet with, are masses of granules of all sizes. These granules, found also in cancerous tumors, have been supposed, by some of the German pathologists, to be nucleoli of larger cells. They are invariably present in epithelial tumors, especially in those of the face, where they very often constitute the largest portion of the tumor. Sometimes we find them enclosed in cysts; sometimes so united as to form the compound granular cell of Bennett. The peculiar tuberculated appearance seen in epithelial tumors of the lip, consists, microscopically almost entirely of granules.

The most interesting element we meet with, are small cells, (*c*) distinctly granulated; some of which, though comparately few, have nuclei. These cells, if acted on by acetic acid, become more distinct; their granular part darkens, and a cell wall can very easily be recognized. What they are, has not as yet been determined; they may be epidermic globules, or very young epithelial cells. Their size varies; they are, usually, about the 1-4000th of an inch in diameter. In tumors of the lip, I have seen them as large as the 1-3200th of an inch. Lebert,* perhaps, means these cells when he speaks of epidermic globes formed within a cell wall by concentric layers of epidermic cells, ("tassement concentriques des feuillets epidermiques;") if, however, he means the globes formed by cells in juxtaposition, they have never been noticed. They are usually round, and might be designated globular cells. I believe them to be, to a certain degree, characteristic of epithelial tumors, and do not recollect ever to have seen them in cancerous tumors; although they might be found in a cutaneous cancer, containing many epidermic cells. We also find fibrous tissue, entering into the composition of epithelial tumors, and forming cysts, which may be empty, or filled with granules, with epithelial cells, or with globular cells. Rokitansky and Bennett suppose these cysts to be formed, and condensed, by the pressure on epithelial cells, where they occur in great masses. These cysts are sometimes seen in tumors just forming. The cells are often held together by very delicate fibrous tissue, analogous to the intercellular substance, already noticed. Sometimes, especially in epithelial tumors of the eyelid, we find fibroplastic cells, and even fibroplastic mother cells;

*Op. cit.

a circumstance, which masks the characteristics of the tumor, and makes it, even under the microscope, a difficult matter to decide, whether it be cancerous or not.

The enlarged papillæ we meet with, are composed of closely arranged epithelial and globular cells, all so compressed as to give to these papillæ, under the microscope, a distinct outline; their real fibrous structure is thought to be preserved beneath.

If we act by chemical agents on the epithelial cells found in these tumors, they are affected in the same way as ordinary epithelial cells are. Acetic acid, whilst it does not affect the older cells, renders the young ones more distinct; especially their nucleus, which remains so until the cell wall gradually disappears. The nucleus is not affected by ammonia; but may be completely dissolved by caustic potassa. Heat and time do not change the character of the cell much. I have kept an epithelial tumor for ten or twelve days in summer without the cells being spoiled. In this respect they differ from cancer cells, which, probably, on account of their high degree of development, lose their distinctness in the space of twenty-four hours. Alcohol darkens the cell; but does not affect it otherwise. By boiling these tumors, we have a gelatinous substance left; by boiling them with liquor potassæ, we get a substance resembling mucus. Simon, who analyzed some scales from the face of a man, which, under the microscope, consisted of epidermic cells, found that on incineration, they left an ash containing carbonate and phosphate of lime and peroxide of iron; the latter was in such abundance, as to communicate a yellow color to the ash. He further states* that ash, yielded by the incineration of the ordinary thickened skin on the hands and feet, is perfectly white, but yet contains a trace of peroxide of iron.

The development of epithelial tumors is as follows: the first symptom the patient observes is a slight roughness, or sometimes a button-like excrescence. He irritates it by picking; it then assumes the shape of a wart, becomes more vascular, and grows lobular, especially in tumors of the lip. The increase is caused by layer after layer of cells forming; the old cells are driven to the top, and form the crust on the tumor. We can very often, in the first stage, see the epidermis separate from the

*P. 661. Simon's Chemistry of Man, translated by Day.

surrounding skin, and from this the papillæ springing. It is not usually accompanied with much pain; but generally with great itching, which is very annoying to the patient, not only physically, but mentally: sudden changes of weather aggravate this itching. Sometimes the tumor becomes so sensitive as to bleed when touched. After a short period it increases rapidly, its base hardens, the surface cracks, and a slow extending, superficial ulcer is established. The part immediately around the tumor hardens also, and often becomes highly inflamed, when the ulceration begins.

One of the most interesting points in connection with these tumors, is to ascertain whether, in any way, the economy is affected, as it is, generally, in cancerous tumors. The economy can only suffer from the extent of the tumor, its location, or the effect it produces upon the mind of the patient; but this affection cannot put an end to life, like real cancerous tumors, from direct morbid action, which produces the disease in another part of the body. Out of eighteen post-mortem examinations recorded by Lebert and Mayor, the disease could not in one single instance, be found in another part of the body. Of these examinations, seven were made on patients who had had epithelial tumors of the lip, two of the face, four of the arm, four of vulva, and one of back of hand. But although it cannot affect the system, we have the cells absorbed, and sent into the neighboring lymphatics. Lebert has seen this twice in the glands near the lip, and once in the arm. I have seen it occurring in a case of tumor of the lip; and it proves, that enlargement of the glands, is not entirely confined to cancerous tumors. It might be alleged, that glands are lined by epithelium, and hence, that these cells were but gland cells. But the cells lining glands are small, and cannot easily be mistaken for epithelial cells, occurring in tumors of the lip. We could also scarcely find globular cells in lymphatics. Sometimes the cells are infiltrated amongst muscular fibre, even amongst bone; but this is very rare. If infiltration into the surrounding muscles has taken place, the tumor is very apt to return, unless the part has been entirely removed.

The duration of epithelial tumors is very different. Those of the lip do not last so long as those in other parts of the body, and ulcerate quicker, owing, perhaps, to the péculiar anatomical tissue of the lip. The patient very seldom consents to have them removed before their last stage. Out of twenty-one cases that I noted the duration of, up to the time of operation, there were:

| | 3–6 *months* | 1 *year* | 2 *years* | 3 *years* | 4 *years* | 7–9 *years* | 22 *years* |
|---|---|---|---|---|---|---|---|
| Lip, | 1 | 1 | 4 | 2 | 1 | 1 | — |
| Face, | 3 | 1 | 1 | — | 1 | — | — |
| Gums, | — | — | — | — | — | 1 | — |
| Tongue, | — | — | — | 1 | — | — | 1 |
| Trunk, | — | — | — | — | — | 2 | — |
| Extremities, | | — | 1 | — | — | — | — |

From this table it appears that they usually grow slowly; differing, in this respect, from real cancer, which in the skin, grows very rapidly. Very often the first wart remains stationary for a long time; one case that came under my notice, had a wart for nine years before the tumor spread. According to some, the wart in tumors of the face may remain stationary for thirty-one years. These tumors occur oftener in the male than in the female, especially those of the lower lip. Tumors of the face, however, are more frequently met with in females. I have noted:

| | *Men.* | *Women.* | *Total.* |
|---|---|---|---|
| Epithelial tumor of the lip, | 11 | — | 11 |
| " " " face, | 3 | 5 | 8 |
| " " " tongue, | 2 | — | 2 |
| " " " gums, | — | 2 | 2 |
| " " " penis, | 1 | — | 1 |
| " " " trunk & extremities, | 2 | 1 | 3 |
| | 19 | 8 | 27 |

In regard to age, it is found to be chiefly a disease of the old.

*Table of 24 Cases.*

| *Age.* | *Lip.* | *Face.* | *Tongue.* | *Trunk.* | *Extremities.* | *Penis.* |
|---|---|---|---|---|---|---|
| 19–23 | 1 | 3 | — | — | 1 | — |
| 23–30 | — | 2 | — | — | — | — |
| 30—40 | — | 1 | — | — | — | — |
| 40—45 | 4 | — | 1 | — | 1 | — |
| 45—50 | — | — | — | — | — | 1 |
| 50—55 | 3 | — | — | — | — | — |
| 60–65 | 1 | — | 1 | — | — | — |
| 73—80 | 2 | 1 | — | 1 | — | — |
| | 11 | 7 | 2 | 1 | 2 | 1 |

It is worthy of remark, that epithelial tumors seldom, if ever, are found to occur amongst negroes.

The general cause of these tumors is local irritation. Many of them result from friction, which destroys the epidermis, and causes it, in consequence, to be secreted in greater quantity. Those on the face are often caused by constant scratching; those on the lip, by the irritation of smoking short pipes, or by intense heat applied to the part. One case I saw, in a shoemaker, occurring on the abdomen, could be clearly attributed to the pressure from a small board, worn whilst working. The epithelial tumors of the scrotum, (chimney-sweep's cancer) are caused by the irritation of the soot; that they, however, may be caused by any irritant, is shown from the fact, that workmen, exposed to the fumes of arsenic, are liable to the same disease. A great many of these tumors are caused by the injudicious use of caustics. Ricord mentions, that phymosis is a predisposing cause to epithelial tumors of the penis.

As regards the diagnosis of epithelial tumors, although it may be difficult in some instances, yet, in a majority of cases, we are able to make it out, by looking at the history of the case, the general appearance of the tumor, its duration, and its manner of growth. Many of these tumors may also be diagnosticated by the microscope, by cutting off small portions, where this can be done without inconvenience, as in some tumors of the lip and penis. If ulceration has begun, a drop of the fluid on the surface, when examined, will often indicate the nature of the tumor.

The prognosis is highly interesting. From the description given, it might appear, that these tumors were entirely benign, aud not in the slightest degree liable to return. But it is not so; they will sometimes return, those on the lower lip especially, if the elements have been infiltrated into the surrounding tissue. Yet the prognosis is by far more favorable, than in cancer of the skin. Their development is so much slower, that it leaves time for active measures to be adopted, and almost all cases, if treated promptly, can be cured. The disease taken out once, may return; but if taken out a second time, will probably not. Out of all the cases I have been able to follow, not one has as yet returned. The return, when it takes place, is entirely local, and must not be sought for in the system of the patient. The best prognosis, according to Lebert, give the epithelial tumors of the body, next those of the lips and penis, and lastly, those of the vulva, which, according to two cases, published by M. Hugier, by producing chronic inflammation of the intestines, caused death.

In the treatment of epithelial tumors, we have but one object in view,—to destroy the part. This can be done sometimes by caustics, or better still, by the knife. Caustics, if at all used, should be very active; they ought, however, never to be employed, except in cases where the part is not accessible to the knife, or where the patient is unwilling to submit to an operation. In such cases, especially in tumors of the face, the internal use of arsenic has been attended with a great deal of success. If excision be performed, we must operate in healthy tissue, and remove every thing at all connected with the diseased spot. The wound usually heals well, and by first intention. It is well in tumors of the lip, to let the patient use glycerin for some time after the operation, to prevent irritation, and to keep the part moist.

A very singular, but unsuccessful mode* of treating tumors of the lip, has recently been tried by Jobert; he tied the artery, vein, and nerve, leading to the part affected, but soon found that it diminished the tumor, without curing it. If the part removed be very extensive, we have to perform a plastic operation, to fill up the gap. In some cases in the extremities, where the patient is rapidly sinking from hectic fever, or loss of blood, amputation is the only resource. To be enabled the better to trace the difference existing between epithelial tumors and cancer of the skin, we will briefly consider the nature of the latter.

*Cancer of the Skin.*

As before stated, not more than 1-6th of the tumors of the skin called cutaneous cancerous tumors, are really cancers. Out of 40 cutaneous tumors, I have only seen 9 cancers; a proportion even larger than usually met with. They were:

| | |
|---|---|
| Cancer of the lip, - - - - - - - - - | 4 cases. |
| " " face, - - - - - - - - - | 2 " |
| " " penis, - - - - - - - - | 1 " |
| " " gums, - - - - - - - - | 2 " |
| Total, | 9 |

*Noticed in Albers's Jahresbericht for 1850.

The skin on the lip and organs of generation is most frequently attacked. According to all modern writers, we find in the skin all the different varieties of cancer. Bock says, he has most frequently met with scirrhus; whilst the usual opinion is, that it is a mixture of encephaloid and scirrhus. Melanotic cancer is very rare. Bennett in his book on Morbid Growths, observation XL., page 91, reports a case of melanotic cancer affecting the skin. The general appearance of cancerous tumors of the skin, is not very marked, from that of epithelial or epidermic tumors. They, however, rarely attain the size of these; never assume the cauliflower arrangement; ulcerate sooner, with a great deal of pain; and develope their peculiar action, at the same time, or shortly afterwards, in a different part of the body. Velpeau mentions a case, where in an autopsy made on a man who died after an operation for the removal of a cancer of the lip, a cancer was found to exist in the liver. Often in cancers of the lip, we have the sub-maxillary gland becoming cancerous.

As regards the microscopical elements of cancerous tumors of the skin, we find cancer cells, free nuclei, fibrous tissue, epithelial-cells and granules. The cancer-cells are of ordinary size; some have double nuclei, some nucleoli. The nuclei themselves are very large and distinct, measuring about the 1-5000th part of an inch. In several instances I found mother-cells. The crust on the surface of the tumor is composed of cancer-cells and epithelial cells, mixed up with blood globules; sometimes the cancer-cells are so mixed with the epithelial-cells, that these latter appear, as if their nuclei were cancer-cells. The fibres in the middle of the tumor, uniting the cells, are usually very strong and dense, much stronger than the fibres in epithelial tumors. The cysts they form are very much of the same kind as those met with in scirrhus of the breast; they are sometimes empty, sometimes filled with cancerous matter. Cancerous tumors of the skin, like epithelial, begin as a small wart, but are usually harder, and of faster growth. They also ulcerate sooner, causing the neighboring lymphatics to swell. Sometimes there are several small separated tumors, each of which is distinctly prominent, and which give to the whole diseased mass, a peculiar lobulated appearance. The ulceration, if it once begin, goes on very rapidly; the ulcer, however, begins from the derma proper, and not, as in epithelial tumors, from the surface; it is also much deeper. According to most observers, cancer of the nose and penis have the most superficial ulceration. If the disease be cut out, it will very often return speedily, sometimes within the lapse of a few months; and the patient either sinks from the return of the cancer in the same spot, or from its affecting some other organ. Lebert gives a very accurate table of 12 cases, in which he was able to trace the period between the first operation and the return of the tumor:

| | | | |
|---|---|---|---|
| 3 Cases lasted from | - - - - - | 3 to 6 | months. |
| 3 " " | - - - - - | 6 to 9 | " |
| 3 " " | - - - - - | 9 to 12 | " |
| 1 " " | - - - - - | 12 to 15 | " |
| 1 " " | - - - - - | 15 to 18 | " |
| 1 " " | - - - - - | 18 to 24 | " |
| 12 | | | |

The average duration of the disease before it terminates fatally, is about two years. In two cases I was able to follow, one died after eleven, the other after fifteen months. Broca mentions a case, which, although operated on five times, terminated fatally in six months. In regard to sex, it seems, that on the whole, men are more liable to be attacked than women. Out of the cases I have noted, there were seven men and two women. The age most liable is the same as in epithelial tumors; it is chiefly from 45 to 75 years. From this it will appear, that the prognosis is the same as that of cancer generally. The treatment usually preferred is to operate, with the hope of the disease not returning speedily, rather than to let the patient have the horrible suffering, of an open ulcerating cancer. If the glands be much involved, it seems to be the best practice not to operate. The patient must be supported by tonics, and by proper exercise and diet.

The great difference then existing, as we have seen, between epithelial or epidermic, and cancerous tumorsof the skin, is:

| | |
|---|---|
| That one is a local disease. | The other constitutional. |
| One grows slowly. | Grows rapidly. |
| Ulcerates superficially. | Ulcerates deeply. |

| | |
|---|---|
| May return; but only as a local affection. | Always returns; sometimes in another spot. |
| Accompanied with little pain. | Always attended with a great deal of pain, |
| Microscopically shows healthy tissue. | Microscopically shows cancer-cells. |
| Prognosis usually favorable. | Prognosis very grave. |
| Local treatment cures it. | Local treatment only palliates it. |

[To be followed by a report of Cases.]

## Figures and Tables

**Fig. 1. f1:**
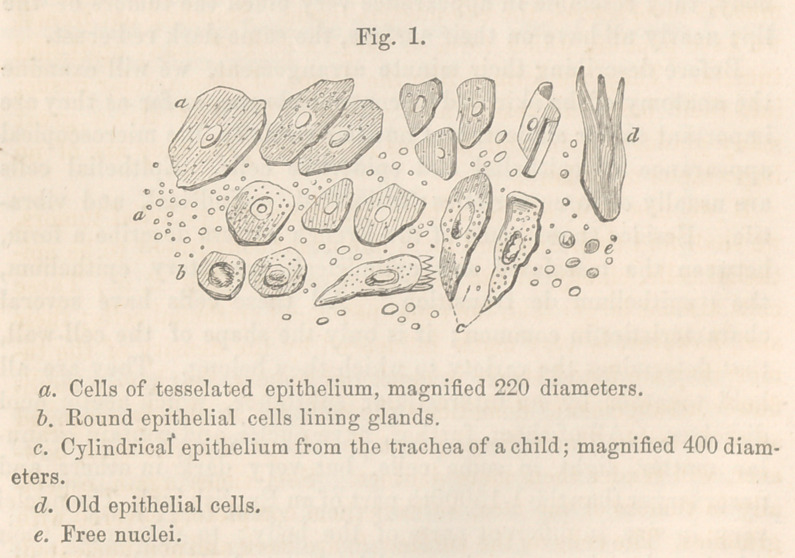


**Fig. 2. f2:**